# How to manage Depression in Dementia and Parkinson’s Disease: A Spanish Consensus

**DOI:** 10.1192/j.eurpsy.2025.160

**Published:** 2025-08-26

**Authors:** L. Aguera-Ortiz

**Affiliations:** Psychiatry, University Hospital 12 de Octubre, Madrid, Spain

## Abstract

**Abstract:**

Psychological and behavioural symptoms are an inherent part of neurodegenerative diseases such as Alzheimer’s disease and other dementias or Parkinson’s disease. Despite the growing research on the subject, there are still large gaps in knowledge about their origin, pathophysiology, diagnosis and treatment. In recent years, various initiatives have been carried out in Spain to improve knowledge, especially on the most controversial issues, of various aspects such as Alzheimer’s disease from the point of view of Psychiatry, the use of antipsychotic drugs or depression in neurodegenerative diseases. The presentation will include data on these national initiatives and will address in more detail two studies using Delphi methodology referring to depression in neurodegenerative diseases. The first one addressed depression in the context of Alzheimer’s disease and other dementias in 53 controversial items regarding risk factors, signs and symptoms, diagnosis and treatment. The second one addresses depression in Parkinson’s disease in 49 controversial issues about the aetiopathological mechanisms, clinical features and connections with motor and non-motor symptoms, diagnostic criteria, and therapeutic options. In both cases, in addition to trying to shed light on etiological and clinical aspects, specific advice is provided on the choice of antidepressant treatment and the particularities linked to its prescription.

**Disclosure of Interest:**

None Declared

